# A Regional Economy’s Resistance to the COVID-19 Shock: Sales Revenues of Micro-, Small-, and Medium-Sized Enterprises in South Korea

**DOI:** 10.1007/s13753-022-00402-9

**Published:** 2022-03-14

**Authors:** Seong-Jin Lee, Joong-Hoo Park, Seung-Min Cha, Donghyun Kim

**Affiliations:** 1 Department of Public Policy and Management, Pusan National University, Busan, 46241 South Korea; 2 Department of Urban Planning and Engineering, Pusan National University, Busan, 46241 South Korea

**Keywords:** COVID-19, Economic resistance, MSMEs, South Korea

## Abstract

The coronavirus disease 2019 (COVID-19) is a global pandemic that has heavily impacted not only the health sector, but also the economic sector in general. Many countries have projected a negative economic impact, and the effect on micro-, small-, and medium-sized enterprises (MSMEs) is predicted to be significantly large. This study estimated the regional resistance of MSME sales revenues and identified the regional economic factors that affect resistance by analyzing South Korea, a country with one of the lowest economic impact projections from COVID-19. Resistance was estimated by comparing sales revenues and changes in resistance observed during the early COVID-19 period to those recorded in the same weeks (weeks 6 to 9) of 2019. The factors that affect regional resistance were determined by conducting robust regression and spatial regression analyses. The results show that the number of confirmed COVID-19 cases, a direct risk factor, is negatively related to regional resilience, while diversity is positively related to regional resistance. To improve the regional resistance against uncertain events, this study recommends increased diversity among regional industrial structures to reduce the duration of the early shock of an unexpected adverse event.

## Introduction

The first case of coronavirus disease 2019 (COVID-19) was reported in December 2019, and COVID-19 was declared a pandemic by the World Health Organization on 13 March 2020. Among its many socioeconomic impacts, the pandemic had a negative economic impact at the regional and global levels. Infectious diseases such as COVID-19 differ from natural hazards and disasters as they cause economic impact rather than physical damage (Ceylan et al. 2020). Severe acute respiratory syndrome coronavirus 1 (SARS-COV) dramatically and quickly, albeit briefly, decreased consumer spending (Siu and Wong 2004; Yang and Chen 2009), while the Middle East respiratory syndrome coronavirus (MERS-COV) caused a decline in both tourism spending (Joo et al. 2019) and retail sales (Jung and Sung 2017).

Previous studies on the economic impact of infectious diseases resulting in disasters—including SARS-COV, MERS-COV, and COVID-19—have mainly focused on the declining gross domestic product (GDP) of a country or the declining employment rate and sales in specific industries (Yang and Chen 2009; Ceylan et al. 2020; Shafi et al. 2020; Fairlie and Fossen 2021). They suggested policy implications that can mitigate the negative effect based on the time series analysis of the economic impact of the diseases. However, these studies were unable to identify forces that can absorb and sustain an unexpected shock such as the COVID-19 crisis. Additionally, the existing studies related to the economic impact of COVID-19 do not reflect the differences in industrial structure at the regional level, as they were conducted at the community or village level or focused on economic impact at the national level.

This study focused on the concept of resistance as the framework of the economic resilience. The concept of resilience, recently utilized in the field of disaster studies, provides a theoretical framework for this research question. In particular, according to the subconcepts constituting the concept of resilience, resistance refers to the extent of withstanding an initial shock (Martin et al. 2016), which will allow us to resolve the limitations of previous studies.

Micro-, small-, and medium-sized enterprises (MSMEs), which are known to have suffered the greatest economic impact from COVID-19 in South Korea in 2020, are the objects of this study. Although South Korea had its first outbreak of COVID-19 before it became a global pandemic, the country’s projected change in GDP was between − 1.2% and − 2.5% in 2020, the lowest among the Organisation for Economic Co-operation and Development (OECD) countries (OECD 2020a). Specifically, the MSMEs of South Korea faced significant sales and employment setbacks due to the COVID-19 shock (OECD 2020b). This study used local administrative units—the basis for MSME economic activity—as the units of analysis to consider the composition and differences of regional economic structures.

This study estimated the regional resistance of MSMEs against COVID-19 shocks and identified the regional economic structure that influences resistance. The regional resistance of MSMEs was estimated by comparing their sales revenues recorded in early 2020, during the first COVID-19 outbreaks (weeks 6–9), to the same weeks of 2019. Robust regression and spatial regression analyses were then performed to identify the regional economic structure that affects resistance. The regional economic structure variables were the agglomeration of manufacturing and service industries and regional industrial diversity.

## Literature Review

The economic impact of COVID-19 has been frequently described in terms of decreasing GDP. Its negative effect on global GDP is projected to approximately range between 2.3 and 4.8% (ADB 2020). The United States and major European countries are projected to experience a 12% drop in GDP, while Japan and South Korea are expected to experience a 6.5% and 4.1% decrease in their GDP, respectively (IMF 2020). The negative economic impact of COVID-19 has been generally found in MSMEs (Bartik et al. 2020; Shafi et al. 2020; Fairlie and Fossen 2021). Compared to large enterprises, MSMEs tend to be financially vulnerable, as their size and resources are insufficient to withstand shocks (Asgary et al. 2020; Bartik et al. 2020). Safety measures such as lockdowns and social distancing are necessary to combat infectious diseases, but they curtail individual consumption (Martin et al. 2020; Hong and Choi 2021). Economic losses and impacts are reflected unevenly across regions (Napierała et al. 2020).

A few studies on the impact of the COVID-19 crisis on MSMEs have focused on the effect of early lockdowns and social distancing measures. Studies generally examined their effect on the industries that have experienced revenue losses. Fairlie and Fossen (2021) found that shutdowns of California in the second quarter of 2020 resulted in an average loss of 17% in sales, with industries such as accommodation being heavily affected by mandatory lockdowns, recording 91% losses of sales. In particular, they found a relationship between COVID-19 patients and sales losses in essential business types and person-to-person contact industries. Motoyama (2020) analyzed cities with industrial structures that may be vulnerable to COVID-19. The study set 2019 figures as baseline values for simulation to study revenue changes and the correlation between population density, land area, and income. Lu et al. (2021) examined the impact of COVID-19 on small- and medium-sized enterprises and their survival. The study found that in 2020, the level of effects differed with respect to industry, and the measures undertaken by the government to control the pandemic are the most important factors for their survival. Prior studies on COVID-19 and its economic impact on MSMEs primarily conducted exploratory analyses on the changes observed in sales. In particular, they focused on the early shocks of COVID-19 and compared them to their corresponding 2019 figures. These studies explained the relationship between the various aspects of the pandemic and MSMEs’ vulnerable characteristics.

Resistance, the main object of this study, refers to the intensity of the reaction to a shock (Martin 2012; Kim and Lim 2016; Martin et al. 2016). The COVID-19 pandemic has acted as an external shock to MSMEs. Economic actors react differently to unexpected external shocks based on their resistance (Martin et al. 2016). Regional resistance, in particular, is affected by the regional economic structure, resources, capabilities, competencies, and local institutions. Studies on regional resistance against recessions have focused on the 2008 global financial crisis with an emphasis on industrial structures. Recent discussions have focused on the diversity in industrial structures (Di Caro 2017; Sedita et al. 2017), the composition of industries (Martin and Gardiner 2019; Giannakis and Bruggeman 2020), and agglomeration economies (Fratesi and Perucca 2018).

## Materials and Methods

We chose the basic levels of South Korea’s local governments*—si* (city), *gun* (county), and *gu* (district)^1^—as the units of analysis. Yeongyang-gun, Sinan-gun, Ulleung-gun, and Gunwi-gun were excluded from the sample because of missing data points. The dependent variable, MSME resistance, was obtained using Eq. 1, following the method introduced by Martin and Gardiner (2019). We compared the average MSME sales revenue recorded in weeks 6 to 9 of 2020 during the COVID-19 outbreak to that of weeks 6 to 9 of 2019.

1 $${\mathrm{RESISTANCE}}_{r}^{t,t-1}=\frac{\Delta {Y}_{r}-\Delta E({Y}_{r})}{|\Delta E\left({Y}_{r}\right)|},$$

where $\Delta E\left({Y}_{r}\right)$ is the expected change in sales ($Y$) in region $r$ during the economic recession of 2020 ($t$) compared to sales of 2019 ($t-1$), given by $\Delta E\left({Y}_{r}\right)=\left(\frac{{Y}_{N}^{t}-{Y}_{N}^{t-1}}{{Y}_{N}^{t-1}}\right)\times {Y}_{r}^{t-1}$.

**Fig. 1:**
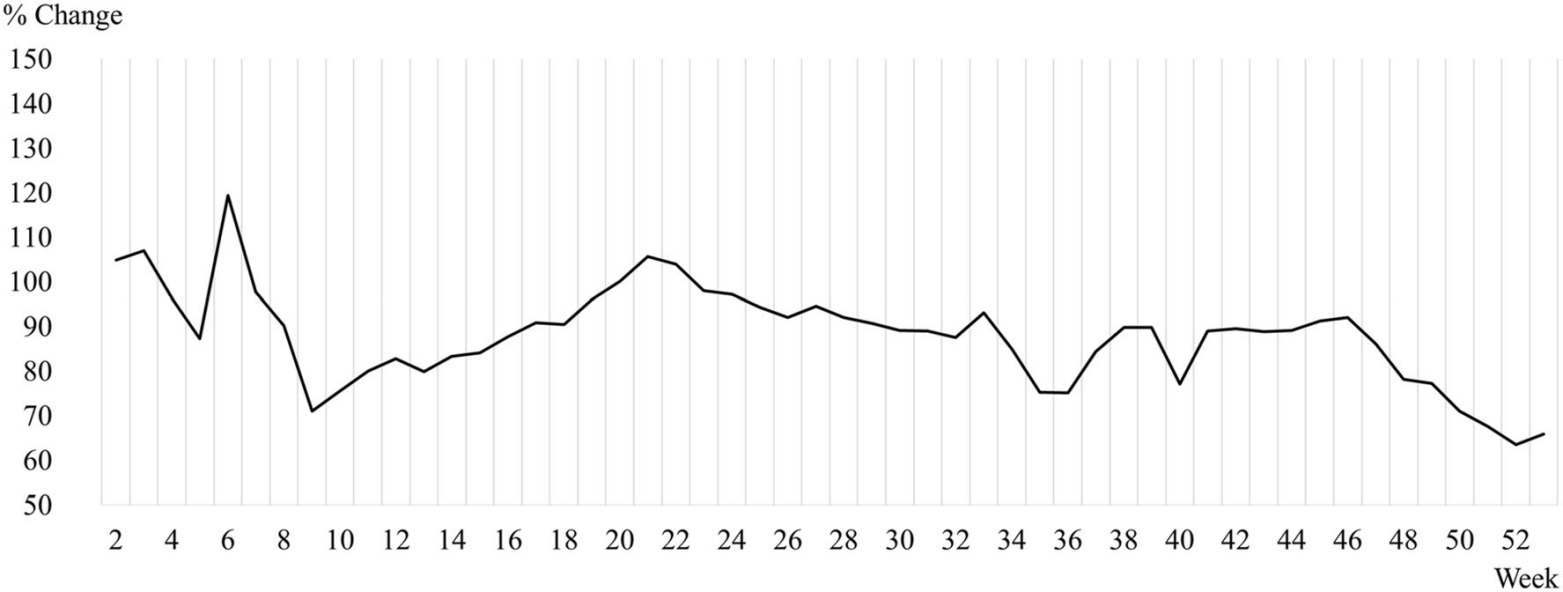
Percentage change of average weekly sales revenue in micro-, small-, and medium-sized enterprises (MSMEs) in South Korea in 2020 compared to 2019. Week 2: 6–12 January; week 3: 13–19 January; week 4: 20–26 January; … week 53: 28 December 2020–3 January 2021

The MSMEs included in this study are defined by Article 2 of the Framework Act on Small and Medium Enterprises, which includes mining, manufacturing, construction, and transportation enterprises with fewer than 10 full-time workers and enterprises in other industries with fewer than 5 full-time workers. The model was constructed using the time periods when MSME sales revenue dropped significantly due to COVID-19 shocks. According to Kim (2021), South Korea experienced three waves during the COVID-19 outbreak. Figure 1 shows the percent changes of MSME average weekly sales revenue in 2020 compared to 2019. The drastic decrease in the four weeks between week 6 and week 9 can be attributed to the COVID-19 shocks observed after its first outbreak. Hence, we considered the resistance against the shocks observed during these four weeks as the dependent variable.

The variables and data used to construct the model are listed in Table 1. For each time period, we used the estimated value of RESISTANCE from Eq. 1. The independent variables consist of the confirmed COVID-19 cases recorded during the first COVID-19 outbreak, the location quotients of manufacturing (LQ_MA), the location quotients of the service sector (LQ_SE), industrial diversity (DIV), population density (POPDEN), and the number of people employed per thousand persons (EMP). We included the LQ of manufacturing, wholesale, and retail industries as agglomeration economy variables, suggesting that these variables are the regional economy’s structural factors that affect shock resistance. Diversity is measured by the entropy index. As we are interested in the resistance of MSME sales revenue against the COVID-19 shock, we included the confirmed COVID-19 cases, population density, and the number of people employed per thousand persons as the control variables. The data from 2020 were used for the confirmed COVID-19 cases and to estimate resistance, while the other variables were constructed using the data from 2018. Equation 2 represents the model used in this study.

2 $${\mathrm{RESISTANCE}}_{r}={\beta }_{0}+{\beta }_{1}{\mathrm{COVID}19}_{r}+{\beta }_{2}{\mathrm{LQ}\_\mathrm{MA}}_{r}+{\beta }_{3}{\mathrm{LQ}\_\mathrm{SE}}_{r}+{\beta }_{4}{\mathrm{DIV}}_{r}+{\beta }_{5}{\mathrm{POPDEN}}_{r}+{\beta }_{6}{\mathrm{EMP}}_{r}+{\epsilon }_{r}.$$

where $r$ is region and $\epsilon$ is error term.

**Table 1:** Variables and data

Variable		Definition	Data
Dependent	RESISTANCE	Resistance in MSME sales revenue observed as the impact of the first outbreak of COVID-19 (weeks 6 to 9, 2020) from Eq. 1	Korea Credit Data (2021)
Independent	COVID-19	COVID-19 confirmed cases in its first outbreak (weeks 6 to 9, 2020)	Korea Disease Control and Prevention Agency (2020)
	LQ_MA	Location quotient of manufacturing employment	Statistics Korea (2018)
	LQ_SE	Location quotient of wholesale and retail employment	Statistics Korea (2018)
	DIV	Industrial diversity measured by the entropy index	Statistics Korea (2018)
	POPDEN	Population density	Statistics Korea (2018)
	EMP	Employment per 1,000 persons	Statistics Korea (2018)

The model of Eq. 2 means that the resistance of MSMEs is treated at the level of individual jurisdiction (spatial unit): *si* (city), *gun* (county), and *gu* (district). Since COVID-19 exhibits a spatial clustering phenomenon (Andrews et al. 2021; Liu et al. 2021), the resistance of MSMEs within the COVID-19 period—the dependent variable of this study—may also have spatial correlation. If the dependent variable has spatial correlation, the linear regression model cannot be BLUE (Best Liear Unbiased Estimator) and the spatial regression model must be considered (Anselin 2002). Therefore, this study used spatial regression models expressed in Eqs. 3 and 4, based on Eq. 2. Two local jurisdictions, Jeju Island and Ulleung Island, were excluded due to the spatial adjacency problem in the spatial regression model. The spatial weight matrix used Queen’s method, which sets the weighted value based on neighboring units sharing any point among contiguity-based weighting calculation methods (Anselin 2005).

3 $$y=\rho {W}_{y}+X\beta +\epsilon , \epsilon \sim N(0,{\sigma }^{2}I)$$

4 $$y=X\beta +\epsilon , \epsilon =\lambda {W}_{\epsilon }+\mu $$

where $y$: $\mathrm{n}\times 1$ matrix of dependent variable (${RESISTANCE}_{r}$); $X$: $\mathrm{n}\times \mathrm{k}$ matrix of independent variable (${COVID19}_{r}, {LQ\_MA}_{r}$, ${LQ\_SE}_{r}$, ${DIV}_{r}, {POPDEN}_{r}$, ${EMP}_{r}$); ${W}_{y}$: $\mathrm{n}\times \mathrm{n}$ spatial weight matrix; $\rho$: spatial lag parameter; $\beta$: $k\times 1$ matrix of regression parameter; $\epsilon$: matrix of error term; $\lambda$: spatial error parameter; ${W}_{\epsilon }$: $\mathrm{n}\times \mathrm{n}$ spatial weight matrix; and $\mu$: matrix of error term.

**Fig. 2:**
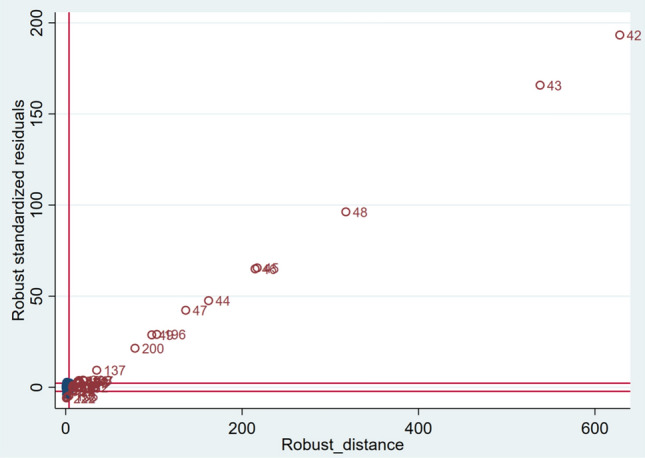
Diagnostic plot of standardized robust residuals versus robust Mahalanobis distance

We chose ordinary least squares (OLS), robust regression, and spatial regression as our methods of analysis. Multiple linear regression analysis generally follows OLS, but in the presence of outliers, the normality assumption may not be valid (Rousseeuw and Leroy 1987; Verardi and Croux 2009). The Shapiro-Wilk W test for normal data returned a W value of 0.988 (*p* = 0.067), which resolved concerns related to normality, and both the Breusch-Pagan and White tests returned values of 2.18 (*p* = 0.1398) and 23.24 (*p* = 0.6722), respectively, ruling out heteroskedasticity. The mean variance inflation factor (VIF) value was 1.47; hence, we did not detect multicollinearity. However, the diagnostic plot (Fig. 2) reveals the existence of vertical outliers and bad leverage. Resolving the outlier problem requires M-estimator, S-estimator, and MM-estimator, which maintain the breakdown point while improving the efficiency by performing a robust regression. Moran’s *I* value was used to confirm spatial correlation as a precondition, using the spatial regression model. For model specification of the spatial regression model, Lagrange multiplier (LM) value, robust LM-lag test, and robust LM-error test were used (Yang 2010).

## Findings

**Fig. 3:**
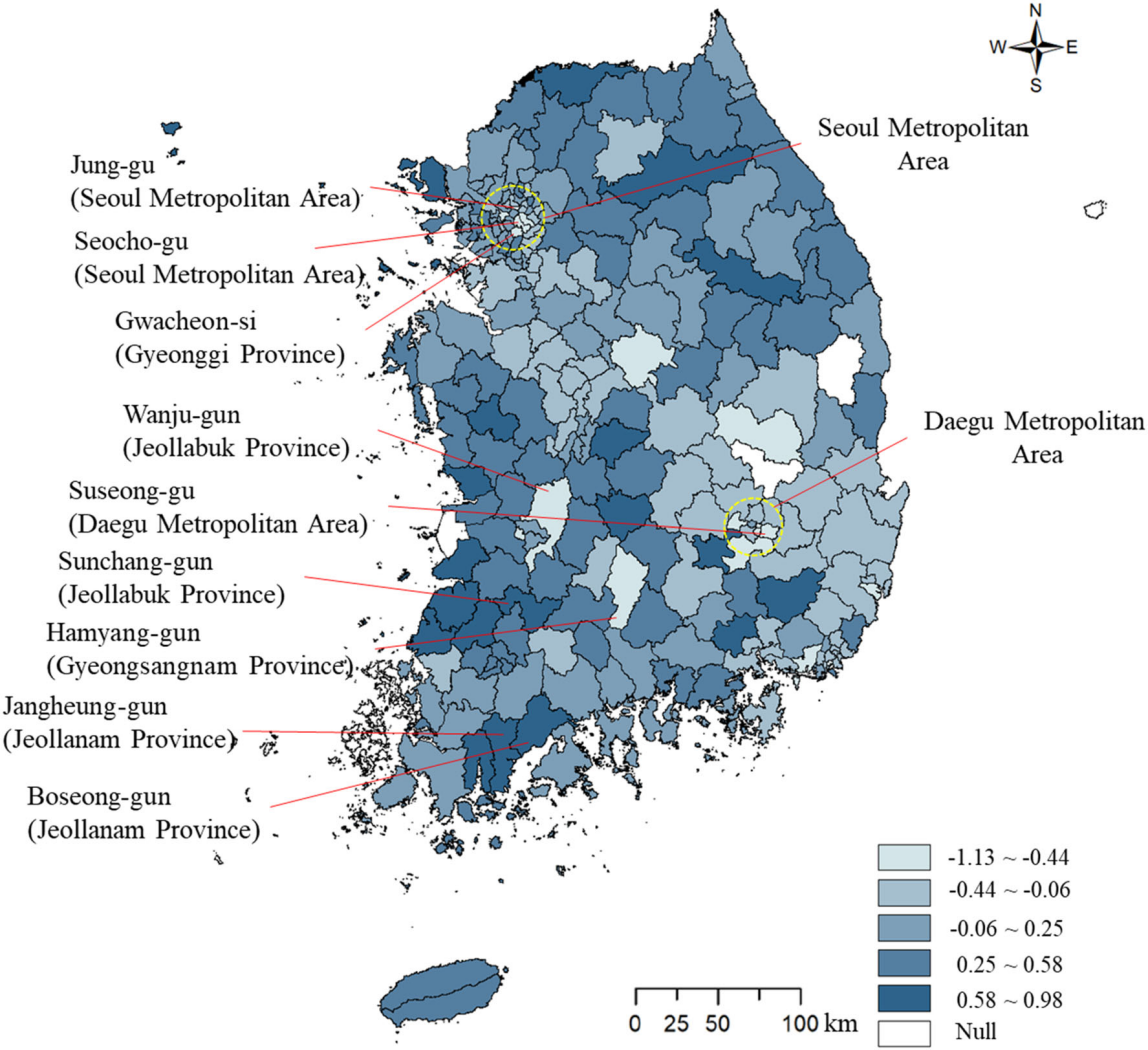
Spatial distribution of micro-, small-, and medium-sized enterprises’ (MSMEs) 2020 regional resistance to early COVID-19 in South Korea. *Si* = city; *Gun* = county; *Gu* = district

The summary statistics of the variables, including MSME resistance to the COVID-19 shock, and the spatial distribution of MSME regional COVID-19 resistance are presented in Table 2 and Fig. 3, respectively. Gwacheon-si, Gyeonggi Province had the lowest resistance, followed by Hamyang-gun, Gyeongsangnam Province; Wanju-gun, Jeollabuk Province; Seocho-gu, Seoul Metropolitan Area; Jung-gu, Seoul Metropolitan Area; and Suseong-gu, Daegu Metropolitan Area. The areas with a high degree of resistance were Jangheung-gun, Jeollanam Province; Sunchang-gun, Jeollabuk Province; and Boseong-gun, Jeollanam Province. The spatial distribution of resistance shows some clusters. High levels of COVID-19 were found in Daegu Metropolitan City’s Nam-gu, Dalseo-gu, Dong-gu, and Suseong-gu, which were the epicenters of the first outbreak.

**Table 2:** Summary statistics

Variable	Mean	Maximum	Minimum	Standard deviation
RESISTANCE	0.121	0.979	− 1.125	0.378
COVID-19	23.487	1148.000	0.000	116.931
LQ_MA	0.989	3.087	0.049	0.756
LQ_SE	0.951	2.086	0.431	0.237
DIV	3.146	3.782	1.413	0.446
POPDEN	4926.314	26,570.406	351.570	5407.150
EMP	438.779	3106.200	186.400	262.820

The results of the OLS, robust regression, and spatial regression analyses are presented in Table 3. The Hausman test finds the Robust-S estimation to be better than the OLS model (${\chi }^{2}$ = 35.704, *p* = 0.000). However, between Robust-S and Robust-MM estimations, the Hausman test fails to provide a definitive answer (${\chi }^{2}$= 10.613, *p* = 0.1011). The Moran’s *I* value of resistance of MSMEs was 0.33 (*p* = 0.01), which was suitable for the application of the spatial regression model. The value of LM was 21.82 (p = 0.01) in the spatial lag model and 15.21 (*p* = 0.01) in the spatial error model. However, the robust LM test value of the spatial error model was not statistically significant, and the robust LM test value of the spatial lag model was 7.95 (*p* = 0.01). Therefore, the spatial lag model was applied as the spatial regression model.

**Table 3:** Ordinary least squares (OLS), robust, and spatial analysis results

Variable	OLS	Robust-M	Robust-S	Robust-MM	Spatial lag model
COVID-19	− 0.000798*** (0.000196)	− 0.000779*** (0.000185)	− 0.0425*** (0.00735)	− 0.0226*** (0.00280)	− 0.000566*** (0.00018)
LQ_MA	0.0245 (0.0495)	0.00906 (0.0555)	0.0762 (0.105)	0.0261 (0.0525)	0.05102 (0.03943)
LQ_SE	− 0.0994 (0.0969)	− 0.176 (0.0900)	− 0.289* (0.115)	− 0.215* (0.0854)	− 0.01751 (0.11109)
DIV	0.219** (0.0696)	0.238** (0.0808)	0.324 (0.217)	0.222** (0.0763)	0.203591*** (0.06531)
POPDEN	− 0.0000150*** (0.00000337)	− 0.0000159*** (0.00000334)	− 0.0000117** (0.00000381)	− 0.0000155*** (0.00000293)	− 0.00000824*** (0.0000043587)
EMP	− 0.000308*** (0.0000880)	− 0.000272** (0.0000835)	− 0.000145 (0.0000739)	− 0.000244*** (0.0000713)	− 0.00026388*** (0.000085842)
Constant	− 0.271 (0.279)	− 0.246 (0.331)	− 0.439 (0.801)	− 0.102 (0.308)	− 0.426239* (0.2441)
Spatial effect ($\rho$)	–	–	–	–	0.37761*** (0.0813631)
${R}^{2}$	0.233	0.239	0.236	0.253	0.316
Efficiency (%)	–	95	28.7	85	–
Scale	0.337	0.302	0.266	0.266	–
*N*	224	224	224	224	222

Standard errors in parentheses

***p < 0.001, **p < 0.01, *p < 0.1

In all models, MSME regional resistance against the COVID-19 shock falls as the number of confirmed COVID-19 cases increases. Although there is some difference from the coefficient value of Robust-S and Robust-MM, it was still statistically significant, as shown in the spatial lag model considering the spatial effect. The confirmation of new cases and the revelation of their movement patterns, often covering long distances, instigated a fear of infection and social contact, resulting in individuals resolving to pursue home isolation, which reduced their consumption and caused an early shock. Hence, MSME resistance against shocks is lower in areas with a high number of confirmed COVID-19 cases.

In Robust-S and Robust-MM models, agglomeration and diversity, that is the factors that affect resistance during a recession, affect resistance differently. Agglomeration had statistically insignificant effects on manufacturing and was found to be positively related to resistance, while agglomeration in wholesale and retail had a negative relationship with resistance. The areas with a high concentration of wholesale and retail industries directly related to consumption showed low resistance. Wholesale and retail industries were directly related to consumption. The low resistance in areas where these industries are concentrated could explain the possibility of a complex relationship between the initial lockdown and social distancing due to COVID-19 and the decrease in consumption brought about by individuals’ psychological behavior. However, in the spatial lag model that considers the spatial effect on resistance of MSMEs, none of the agglomeration variables were statistically significant. This means that if the situation where COVID-19 affects the resistance of MSMEs in adjacent spaces through epidemiological transmission is considered, the regional industrial agglomeration cannot specify the relationship with the resistance of MSMEs in the region. The Robust-S and Robust-MM models assume that the phenomenon of disaster is independent per a spatial unit. This means that when the spatial dependences of disaster were not considered, the agglomeration of the industry related to the object of resistance can affect it. However, disasters such as COVID-19 that cluster with surrounding areas and show their socioeconomic impacts mean that the relationship with the surrounding areas is more important than the industrial agglomeration characteristics of the area.

Similar to previous studies (Di Caro 2017; Sedita et al. 2017), diversity was found to be positively related to resistance in all models. High levels of diversity in regional industrial structures mean that other substitutable industries can absorb some of the effects. It appears that MSME resistance to COVID-19 shocks is affected by the diversity in regional industrial structures. The control variables population density, and the number of people employed per thousand individuals, are negatively related in all models. Population density indicates an area’s level of urbanization, and the number of people employed per thousand persons indicates the size of the area’s economy. The negative relationship between both the variables suggests that urbanized areas and those with large economies exhibit low resistance. For the Robust-S and Robust-MM models, the coefficient of the COVID-19 variable denotes a decrease in MSME resistance with an increase of one confirmed COVID-19 case. The ratio of the estimated coefficient value to the standard deviation of the resistance indicator is 5% to 10%, which implies that the initial shock of COVID-19 had a large impact.

## Discussion

This study helps us understand the differences in the regional economic impact of disasters, for example, the unexpected and sudden impacts of infectious diseases such as COVID-19. Governments in many countries have used social distancing and lockdown policies (Castex et al. 2021; Glogowsky et al. 2021; Woskie et al. 2021) to contain the spread of the disease. The initial impact of the unexpected shocks and unprecedented policies appears to have been large, and this study focuses on the resistance response to this shock. The resistance of MSMEs associated with the low- and middle-income classes has been negatively affected due to COVID-19, such that many governments are implementing financial subsidies for MSMEs. People with low socioeconomic status are less resilient and more likely to fall back into poverty (Ur Rahman et al. 2021). Low- and middle-income class people working in MSMEs are also likely to be less resilient and to have a worse socioeconomic status than in their pre-COVID-19 situation. In order to respond to the shock experienced by low socioeconomic status groups of MSMEs, policies that can increase income in the short term are needed (Shafi et al. 2021). Subsidies for employment and business are also required to minimize vulnerabilities (Ur Rahman et al. 2021). As well as for MSMEs to respond more sustainably, it is necessary to ensure diversity in the local industrial structure, as indicated by the results of this study.

This study also showed that the role of industrial diversity, which is known to have positive externalities in regional economic growth, plays a positive role in the regional economic resistance to the unexpected shock of COVID-19. The positive role of industrial diversity in regional economic growth was explained by the knowledge externalities of the interactions performed among workers with diverse industries, jobs, and skills in the region (Boschma 2005; Frenken et al. 2007; Boschma et al. 2014). Such industrial diversity plays a positive role not only in growth, but also in answer to shock. Diversity provides redundancy to the economic system through overlapping and complementary functions and plays a positive role in absorbing the impact of a shock (Martin and Sunley 2015). A more diverse industry structure showed better employment performance with respect to the impact of the Great Recession (Brown and Greenbaum 2017), and economic diversity was an effective part of the resilience related to regional recovery and growth, even during and after natural hazard-related disasters such as floods. The fact that regional industrial diversity plays a positive role in the resistance of MSMEs to the shock of COVID-19 is attributed to the existence of multiple networks between local MSMEs and other industries and redundancy, which perform a complementary function in mitigating the impact of shocks.

This study has two main implications. First, industrial policies that encourage diversity are required to reduce the regional impact of unexpected shocks on MSMEs. Although the confirmed cases directly related to COVID-19 shocks caused related changes and had a negative effect on resistance, the magnitude of effect was less than diversity. Hence, diversity in a region’s industrial structure is more important for resistance than the direct effects of a variable related to shocks. Studies on recessions have shown that a region’s industrial structure diversity has positive effects on resistance and recovery, which was also found to be true in the context of COVID-19. However, MSMEs are structurally vulnerable to unexpected shocks and various types of recession. Our results emphasize the need for mid- to long-term plans that encourage regional diversity, rather than the implementation of plans to support MSMEs directly, to improve their regional resistance.

Second, in the expectation that the future will entail various threats with highly uncertain effects, alternative policies are needed to establish a more resilient structure by reducing the impact of early shocks. The impact of early shocks is driven by panic created by misinformation and related behaviors, which have various ripple effects. During the early shock of COVID-19, there was a fear of infection, an increase in misinformation, a decrease in consumption, and consequently, there were decreases in MSME revenues. Our results show that MSME revenues did not change as dramatically during the later COVID-19 outbreaks observed in 2020 compared to the early shock. Because MSMEs have weak financial structures and resources, their survival may depend on the length of the early shock. Thus, this study suggests that for the shocks that entail uncertainty and unexpectedness, such as COVID-19, reducing the early shock duration and providing correct information are essential to improving resistance.

## Conclusion

This study examined the South Korean MSMEs most affected by COVID-19 by estimating their regional resistance to early COVID-19 shocks and identifying the regional economic factors affecting regional resistance. The spatial distribution of MSME regional resistance was partly concentrated in the southwest and northeast areas of South Korea, and spatial correlations of MSME regional resistance were identified. The increase in the number of confirmed COVID-19 cases showed a negative relationship with regional resistance. Among the regional economic factors that affect MSME regional resistance, diversity was the most positively related, while the agglomerations of the wholesale and retail industry sectors, consisting of many MSMEs, were negatively related. The agglomeration of the manufacturing industry was positively related to regional resistance but was not statistically significant. Densely populated urban area and the number of confirmed COVID-19 cases were negatively related to MSME regional resistance.

This study has limitations. While it addresses the outliers by utilizing robust regression, this is a limitation as the model does not consider business fluctuations, the second and third waves of the COVID-19 outbreaks through time series data, and the relationships of MSMEs with large enterprises. To include business fluctuations, the data on MSME revenues recorded before 2019 are required. However, these data are limited, and were not included in the model. Government relief programs and safety measures have changed over time, with the implementation of varying degrees of social distancing and lockdown measures. These factors must be controlled to include shocks from the second and third pandemic waves in 2020. However, because it is difficult to compare the different relief policies and measures intended to control infectious diseases, they were excluded from this study. Finally, it is important to study the change in MSME revenues in relation to not only individual consumers, but also large enterprises. However, because of insufficient data to study the relationships between MSMEs and large enterprises, they were excluded from this study. Therefore, the results of this study should be interpreted such that there are relevant linkages between resistance, COVID-19, and the related economic variables. However, no causal relationship was determined.

## Footnotes

1 *si* (city), *gun* (county), and *gu* (district) are the basic spatial units of local government below metropolitan area and province in South Korea.

## References

[CR1] ADB (Asian Development Bank) (2020). Asian development outlook: What drives innovation in Asia? Specific topic: The impact of the coronavirus outbreak—An update: Highlights.

[CR2] Andrews, M.R., K. Tamura, J.N. Best, J.N. Ceasar, K.G. Batey, T.A. Kearse, Jr., L.V. Allen III, Y. Baumer, et al. 2021. Spatial clustering of county-level COVID-19 rates in the U. S. International Journal of Environmental Research and Public Health 18(22): Article 12170. 10.3390/ijerph182212170 PMC8622138 34831926

[CR3] Anselin L (2002). Under the hood issues in the specification and interpretation of spatial regression models. Agricultural Economics.

[CR4] Anselin, L. 2005. Exploring spatial data with GeoDa™: A workbook. Santa Barbara, CA: Center for Spatially Integrated Social Science, University of California. https://geodacenter.github.io/docs/geodaworkbook.pdf. Accessed 1 Mar 2022.

[CR5] Asgary A, Ozdemir AI, Özyürek H (2020). Small and medium enterprises and global risks: Evidence from manufacturing SMEs in Turkey. International Journal of Disaster Risk Science.

[CR6] Bartik, A.W., M. Bertrand, Z.B. Cullen, E. Glaeser, M. Luca, and C.T. Stanton. 2020. How are small businesses adjusting to COVID-19? Early evidence from a survey. NBER (National Bureau of Economic Research) Working Paper No. 26989. 10.3386/w26989. Accessed 1 Mar 2022.

[CR7] Boschma R (2005). Proximity and innovation: A critical assessment. Regional Studies.

[CR8] Boschma R, Heimeriks G, Balland P-A (2014). Scientific knowledge dynamics and relatedness in biotech cities. Research Policy.

[CR9] Brown L, Greenbaum RT (2017). The role of industrial diversity in economic resilience: An empirical examination across 35 years. Urban Studies.

[CR10] Castex G, Dechter E, Lorca M (2021). COVID-19: The impact of social distancing policies, cross-country analysis. Economics of Disasters and Climate Change.

[CR11] Ceylan RF, Ozkan B, Mulazimogullari E (2020). Historical evidence for economic effects of COVID-19. European Journal of Health Economics.

[CR12] Di Caro P (2017). Testing and explaining economic resilience with an application to Italian regions. Papers in Regional Science.

[CR13] Fairlie R, Fossen FM (2021). The early impacts of the COVID-19 pandemic on business sales. Small Business Economics.

[CR14] Fratesi U, Perucca G (2018). Territorial capital and the resilience of European regions. Annals of Regional Science.

[CR15] Frenken K, Van Oort F, Verburg T (2007). Related variety, unrelated variety and regional economic growth. Regional Studies.

[CR16] Giannakis E, Bruggeman A (2020). Regional disparities in economic resilience in the European Union across the urban–rural divide. Regional Studies.

[CR17] Glogowsky, U., E. Hansen, and S. Schächtele. 2021. How effective are social distancing policies? Evidence on the fight against COVID-19. PLOS ONE 16: Article e0257363. 10.1371/journal.pone.0257363 PMC8457454 34550995

[CR18] Hong, S., and S.-H. Choi. 2021. The urban characteristics of high economic resilient neighborhoods during the COVID-19 pandemic: A case of Suwon, South Korea. Sustainability 13(9): Article 4679.

[CR19] IMF (International Monetary Fund) (2020). World economic outlook update, June 2020: A crisis like no other, an uncertain recovery.

[CR20] Joo H, Maskery BA, Berro AD, Rotz LD, Lee Y-K, Brown CM (2019). Economic impact of the 2015 MERS outbreak on the Republic of Korea’s tourism-related industries. Health Security.

[CR21] Jung, E., and H. Sung. 2017. The influence of the Middle East Respiratory Syndrome outbreak on online and offline markets for retail sales. Sustainability 9(3): Article 411.

[CR22] Kim D (2021). Visualizing the regional patterns of two crises: The COVID-19 outbreak and decreasing MSME sales during three different phases of 2020 in Korea. Environment and Planning A: Economy and Space.

[CR23] Kim, D., and U. Lim. 2016. Urban resilience in climate change adaptation: A conceptual framework. Sustainability 8(4): Article 405.

[CR24] Korea Credit Data. 2021. Data portal. https://forum.cashnote.kr/data_portal. Accessed 27 Jul 2021.

[CR25] Korea Disease Control and Prevention Agency. 2020. Coronavirus (COVID-19), Republic of Korea. http://ncov.mohw.go.kr/en. Accessed 1 Mar 2022.

[CR26] Liu, M., M. Liu, Z. Li, Y. Zhu, Y. Liu, X. Wang, L. Tao, and X. Guo. 2021. The spatial clustering analysis of COVID-19 and its associated factors in mainland China at the prefecture level. Science of the Total Environment 777: Article 145992.

[CR27] Lu, L., J. Peng, J. Wu, and Y. Lu. 2021. Perceived impact of the COVID-19 crisis on SMEs in different industry sectors: Evidence from Sichuan, China. International Journal of Disaster Risk Reduction 55: Article 102085. 10.1016/j.ijdrr.2021.102085 PMC9188648 35719701

[CR28] Martin A, Markhvida M, Hallegatte S, Walsh B (2020). Socio-economic impacts of COVID-19 on household consumption and poverty. Economics of Disasters and Climate Change.

[CR29] Martin R (2012). Regional economic resilience, hysteresis and recessionary shocks. Journal of Economic Geography.

[CR30] Martin R, Gardiner B (2019). The resilience of cities to economic shocks: A tale of four recessions (and the challenge of Brexit). Papers in Regional Science.

[CR31] Martin R, Sunley P (2015). On the notion of regional economic resilience: Conceptualization and explanation. Journal of Economic Geography.

[CR32] Martin R, Sunley P, Gardiner B, Tyler P (2016). How regions react to recessions: Resilience and the role of economic structure. Regional Studies.

[CR33] Motoyama Y (2020). What kind of cities are more vulnerable during the COVID-19 crisis?. Local Development & Society.

[CR34] Napierała, T., K. Leśniewska-Napierała, and R. Burski. 2020. Impact of geographic distribution of COVID-19 cases on hotels’ performances: Case of Polish cities. Sustainability 12(11): Article 4697.

[CR35] OECD (Organization for Economic Co-Operation and Development) (2020). OECD Economic Outlook 2020(1).

[CR36] OECD (Organization for Economic Co-Operation and Development) (2020). OECD economic survey: Korea 2020.

[CR37] Rousseeuw PJ, Leroy AM (1987). Robust regression and outlier detection.

[CR38] Sedita SR, De Noni I, Pilotti L (2017). Out of the crisis: An empirical investigation of place-specific determinants of economic resilience. European Planning Studies.

[CR39] Shafi, M., J. Liu, and W. Ren. 2020. Impact of COVID-19 pandemic on micro, small, and medium-sized enterprises operating in Pakistan. Research in Globalization 2: Article 100018.

[CR40] Shafi, M., J. Liu, J. Deng, I.U. Rahman, and X. Chen. 2021. Impact of the COVID-19 pandemic on rural communities: A cross-sectional study in the Sichuan Province of China. BMJ Open 11: Article e046745. 10.1136/bmjopen-2020-046745 PMC8359857 34376445

[CR41] Siu A, Wong YCR (2004). Economic impact of SARS: The case of Hong Kong. Asian Economic Papers.

[CR42] Statistics Korea. 2018. Korea Statistical Information Service. https://kosis.kr/index/index.do. Accessed 27 Jul 2021.

[CR43] Ur Rahman, I., J. Deng, J. Liu, and M. Shafi. 2021. Socio-economic status, resilience, and vulnerability of households under COVID-19: Case of village-level data in Sichuan Province. PLOS ONE 16: Article e0249270. 10.1371/journal.pone.0249270 PMC8084142 33914745

[CR44] Verardi V, Croux C (2009). Robust regression in Stata. The Stata Journal: Promoting Communications on Statistics and Stata.

[CR45] Woskie, L.R., J. Hennessy, V. Espinosa, T.C. Tsai, S. Vispute, B.H. Jacobson, C. Cattuto, L. Gauvin, et al. 2021. Early social distancing policies in Europe, changes in mobility & COVID-19 case trajectories: Insights from spring 2020. PLOS ONE 16: Article e0253071. 10.1371/journal.pone.0253071 PMC8244916 34191818

[CR46] Yang H-Y, Chen K-H (2009). A general equilibrium analysis of the economic impact of a tourism crisis: A case study of the SARS epidemic in Taiwan. Journal of Policy Research in Tourism, Leisure and Events.

[CR47] Yang Z (2010). A robust LM test for spatial error components. Regional Science and Urban Economics.

